# *YAP1-MAML2* fusion in a pediatric NF2-wildtype intraparenchymal brainstem schwannoma

**DOI:** 10.1186/s40478-022-01423-7

**Published:** 2022-08-19

**Authors:** Matthias A. Karajannis, Bryan K. Li, Mark M. Souweidane, Benjamin Liechty, JinJuan Yao, Jamal K. Benhamida, Tejus A. Bale, Marc K. Rosenblum

**Affiliations:** 1 Pediatric Neuro-Oncology Service, Department of Pediatrics, Memorial Sloan Kettering Cancer Center, New York, NY 10065 USA; 2 Department of Neurosurgery, Weill Cornell Medicine and Memorial Sloan Kettering Cancer Center, New York, NY USA; 3 Department of Pathology, Weill Cornell Medicine, New York, NY USA; 4 Department of Pathology, Memorial Sloan Kettering Cancer Center, New York, NY USA

**Keywords:** Schwannoma, Pediatric, Mastermind like transcriptional coactivator 2 (MAML2), Yes1 associated transcriptional regulator (YAP1)

## Abstract

Biallelic inactivation of NF2 represents the primary or sole oncogenic driver event in the vast majority of schwannomas. We report on a four-year-old female who underwent subtotal resection of a right medullary intraparenchymal schwannoma. RNA sequencing revealed an in-frame fusion between exon 5 of *YAP1* and exon 2 of *MAML2*. *YAP1-MAML2* fusions have previously been reported in a variety of tumor types, but not schwannomas. Our report expands the spectrum of oncogenic YAP1 gene fusions an alternative to NF2 inactivation to include sporadic schwannoma, analogous to what has recently been described in NF2-wildtype pediatric meningiomas. Appropriate somatic and germline molecular testing should be undertaken in all young patients with solitary schwannoma and meningioma given the high prevalence of an underlying tumor predisposition syndrome. In such patients, the identification of a somatic non-NF2 driver alteration such as this newly described YAP1 fusion, can help ascertain the diagnosis of a sporadic schwannoma.

## Background

Schwannomas are benign peripheral nerve sheath tumors that arise sporadically or in the context of inheritable tumor predisposition; i.e., neurofibromatosis type 2 (NF2) or schwannomatosis. Biallelic inactivation of *NF2* represents the primary or sole oncogenic driver event in the vast majority of schwannomas; in addition, *SH3PXD2A-HTRA1* fusions have been recently identified as an alternative oncogenic driver in a subset of sporadic schwannomas [1, 2]. Among pediatric and young adult patients with newly diagnosed solitary schwannoma, up to 30% will ultimately be diagnosed with an inheritable tumor predisposition syndrome, i.e. NF2 or less commonly, schwannomatosis. However, accurate determination of germline status can be difficult given the high prevalence of mosaicism associated with false-negative germline testing results [3].

## Case presentation

**Fig. 1:**
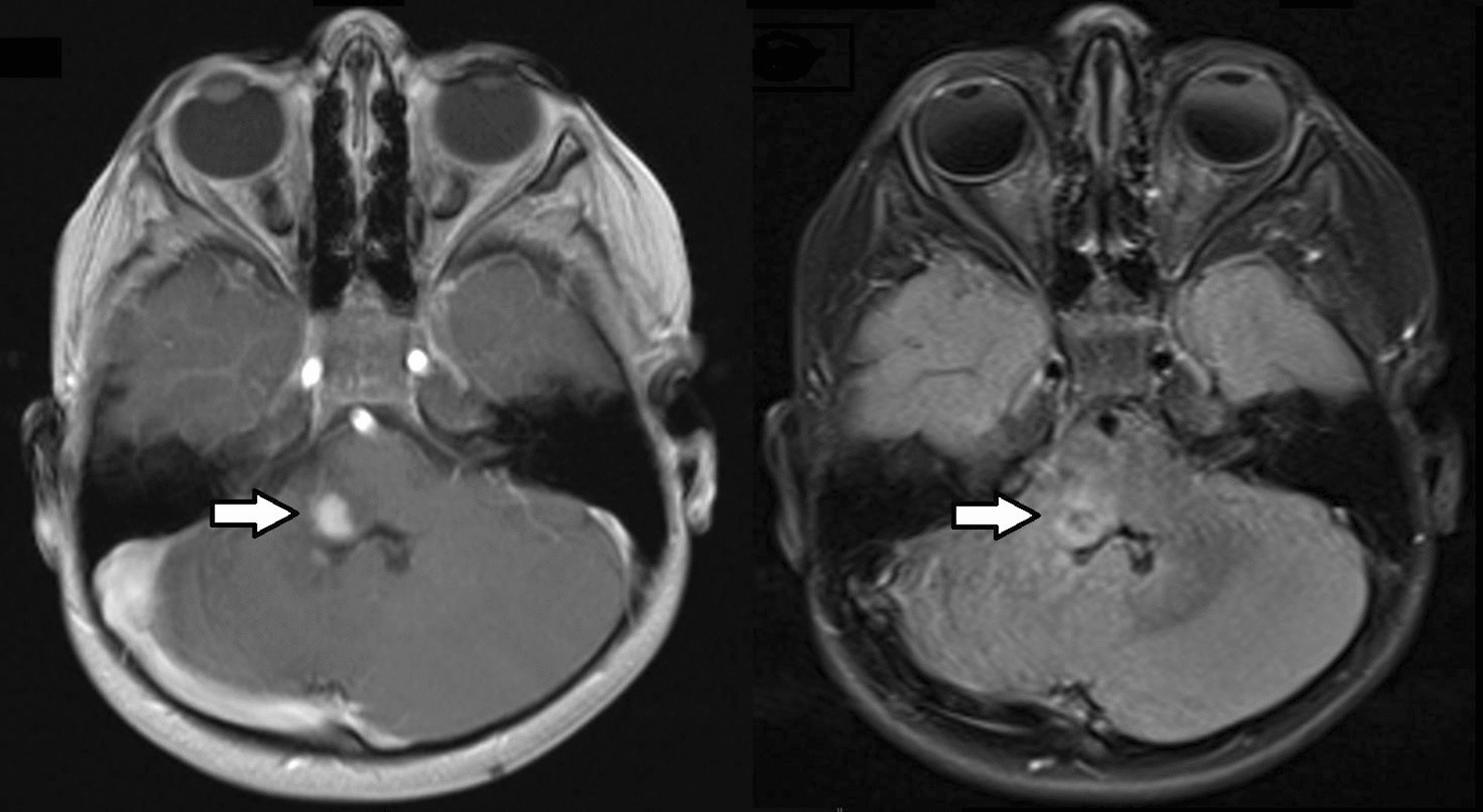
T1-weighted post-contrast (left panel) and T2/FLAIR-weighted (right panel) magnetic resonance images revealing a partially contrast-enhancing intra-parenchymal right medullary tumor (arrows). The tumor involved the right lateral aspect of the inferior pons, brachium pontis and ventral cerebellum

A four-year-old female underwent subtotal resection of a right medullary schwannoma. The tumor was identified on magnetic resonance imaging (MRI) obtained for progressive head tilt (Fig. 1). Past medical history was notable for developmental delay and facial asymmetry (hemifacial microsomia). A prior MRI performed at age 18 months showed cerebellar hypoplasia, but no tumor. A comprehensive clinical genetic workup including targeted and whole exome sequencing of the germline was negative. Most recent follow-up MRI of the brain eight months after diagnosis showed stable residual disease.

**Fig. 2:**
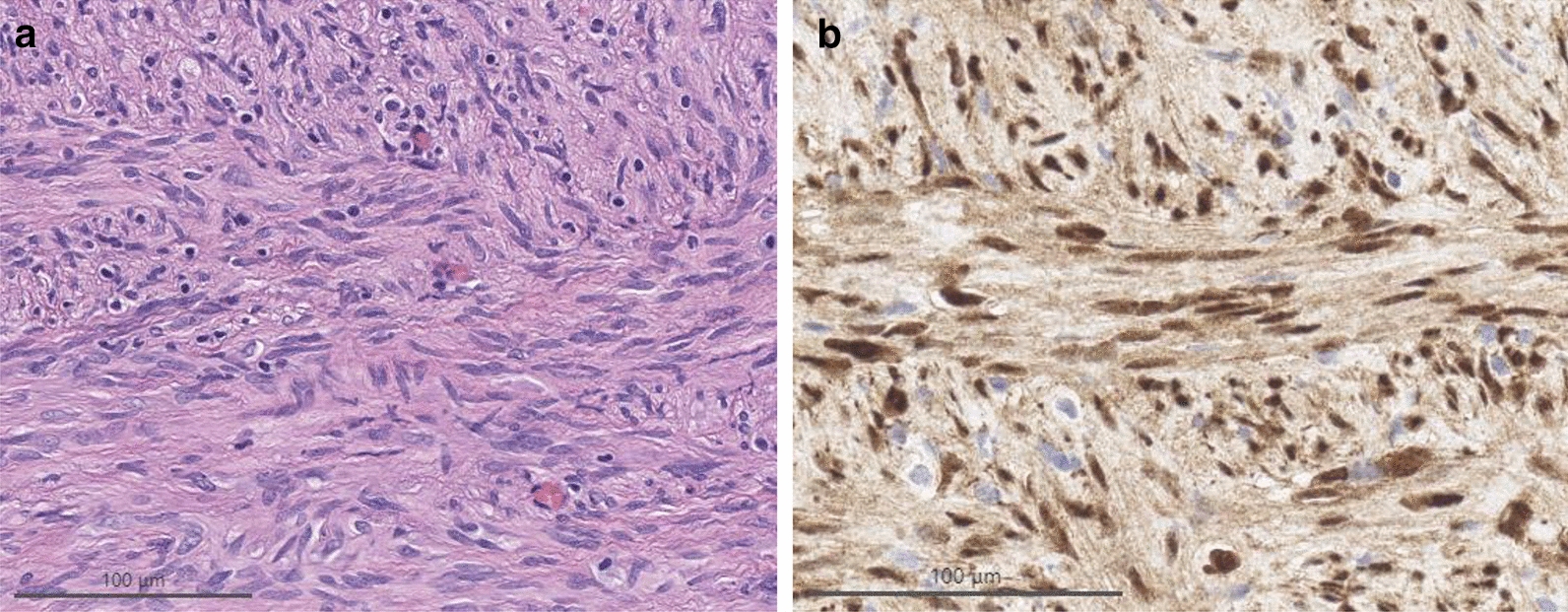
Hematoxylin and eosin staining of the tumor (**a**) and immunohistochemistry for YAP1 (**b**)

Histologic examination disclosed a neoplasm composed of monomorphous spindle cells in tight, interlacing fascicles (Fig. 2a), without Verocay bodies or Antoni B areas. Devoid of mitotic activity, tumor cells focally dissected into adjoining neuroparenchyma along blood vessels. Immunohistochemical studies showed the lesion to be rich in collagen IV, with tumor cells being negative for GFAP, EMA and SSTR2A, while expressing S100 protein (cytoplasmic/nuclear) and SOX10 (nuclear).

**Fig. 3:**
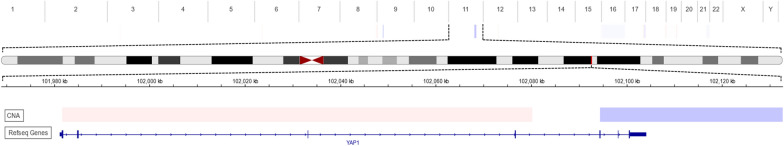
DNA copy number profile derived from paired targeted next-generation sequencing analysis of tumor and matched normal sample, revealing a relatively flat DNA copy number profile with focal genomic gains (depicted in red) and losses (depicted in blue) at chromosome 11q including the *YAP1* locus. CNA: copy number alterations

**Fig. 4:**
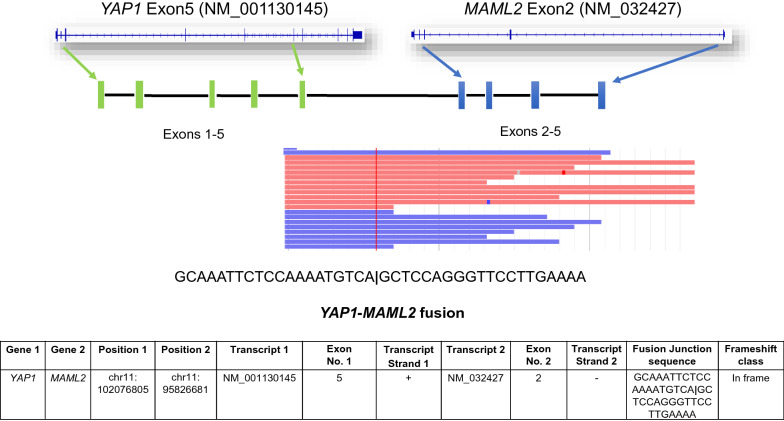
RNA sequencing using Anchored Multiplex PCR revealing an in-frame fusion between exon 5 of YAP1 and exon 2 of MAML2

DNA methylation profiling [4] with the Heidelberg brain tumor classifier version 11b6 revealed a match to the methylation class schwannoma with a calibrated score of 0.97. Paired targeted next-generation sequencing analysis of tumor and matched normal sample [5] was negative for somatic mutations as well as structural variants, and revealed a relatively flat DNA copy number profile with focal genomic gains and losses at chromosome 11q including the *YAP1* locus (Fig. 3) [6]. RNA sequencing using Anchored Multiplex PCR [7] revealed an in-frame fusion between exon 5 of *YAP1* and exon 2 of *MAML2* (Fig. 4). *YAP1-MAML2* fusions have been reported in NF2-wild type meningioma [8] and other cancers [9], but not schwannomas. As seen in all N-terminal YAP1 fusions reported to date, the fusion detected in our patient retains the TEAD transcription factor binding domain of YAP1, along with the nuclear localization sequence and transactivation domain of MAML2. The resulting fusion protein is resistant to inhibitory signaling of the Hippo tumor suppressor pathway by constitutive nuclear localization and resistance to proteasomal degradation [10]. In keeping with these observations, we confirmed strong nuclear localization of YAP1, as well as weaker cytoplasmic labelling in our patient’s tumor by immunohistochemistry (Fig. 2b).

## Discussion and conclusions

Our finding of an oncogenic *YAP1-MAML2* fusion in an NF2 wild-type schwannoma supports the notion that canonical Hippo signaling through the effectors YAP/TAZ is required for schwannomagenesis [11]. Intraparenchymal schwannomas including brainstem schwannomas are rare, and molecularly not well characterized [12].

Among pediatric and young adult patients with solitary schwannoma or meningioma, up to 30% and 50%, respectively, will have an identifiable genetic predisposition, most commonly NF2 [3]. Accordingly, appropriate clinical screening examinations and molecular genetic testing of tumor and germline are recommended for all young patients with solitary schwannoma or meningioma. However, a diagnosis of NF2 or schwannomatosis can be difficult to rule out in patients with negative germline testing due to the high prevalence of mosaicism [13]. In such patients, the identification of a somatic non-NF2 driver alteration such as a *YAP1* fusion, can help ascertain the diagnosis of a sporadic schwannoma or meningioma with confidence, and may obviate the need for further genetic or clinical testing to rule out an inheritable tumor predisposition syndrome.

## Data Availability

All data generated or analyzed during this study are included in this published article.

## Abbreviations

EMA: Epithelial membrane antigen
GFAP: Glial fibrillary acidic protein
HTRA1: HtrA serine peptidase 1
MAML2: Mastermind like transcriptional coactivator 2
MRI: Magnetic resonance imaging
NF2: NF2, moesin-ezrin-radixin like (MERLIN) tumor suppressor
S100: S100 calcium binding protein
SH3PXD2A: SH3 and PX domains 2A gene
SOX10: SRY-box transcription factor 10
SSTR2A: Somatostatin receptor subtype 2A
YAP1: Yes1 associated transcriptional regulator
